# Longitudinal evaluation the pulmonary function of the pre and postoperative periods in the coronary artery bypass graft surgery of patients treated with a physiotherapy protocol

**DOI:** 10.1186/1749-8090-6-62

**Published:** 2011-04-27

**Authors:** Adalgiza M Moreno, Renata RT Castro, Pedro PS Sorares, Mauricio Sant' Anna, Sergio LD Cravo, Antônio CL Nóbrega

**Affiliations:** 1 Post-graduate Program in Cardiovascular Sciences, Fluminense Federal University, Niteroi, RJ, Brazil; 2 Department of Physiology and Pharmacology, Fluminense Federal University, Rio de Janeiro, Niteroi, RJ, Brazil; 3 Federal University of Rio de Janeiro, RJ, Brazil; 4 Department of Physiology, Universidade Federal de São Paulo, SP, Brazil

## Abstract

**Background:**

The treatment of coronary artery disease (CAD) seeks to reduce or prevent its complications and decrease morbidity and mortality. For certain subgroups of patients, coronary artery bypass graft surgery (CABG) may accomplish these goals. The objective of this study was to assess the pulmonary function in the CABG postoperative period of patients treated with a physiotherapy protocol.

**Methods:**

Forty-two volunteers with an average age of 63 ± 2 years were included and separated into three groups: healthy volunteers (n = 09), patients with CAD (n = 9) and patients who underwent CABG (n = 20). Patients from the CABG group received preoperative and postoperative evaluations on days 3, 6, 15 and 30. Patients from the CAD group had evaluations on days 1 and 30 of the study, and the healthy volunteers were evaluated on day 1. Pulmonary function was evaluated by measuring forced vital capacity (FVC), maximum expiratory pressure (MEP) and Maximum inspiratory pressure (MIP).

**Results:**

After CABG, there was a significant decrease in pulmonary function (p < 0.05), which was the worst on postoperative day 3 and returned to the preoperative baseline on postoperative day 30.

**Conclusion:**

Pulmonary function decreased after CABG. Pulmonary function was the worst on postoperative day 3 and began to improve on postoperative day 15. Pulmonary function returned to the preoperative baseline on postoperative day 30.

## Background

The treatment of coronary artery disease (CAD) seeks to reduce or prevent its complications and decrease morbidity and mortality. For certain subgroups of patients, coronary artery bypass graft surgery (CABG) may accomplish these goals [1].

Pulmonary dysfunction and associated complications are the major cause of morbidity and mortality in the period following cardiac CABG surgery [2]. The impairment of pulmonary function has multiple causes, including the use of a sternotomy, pleurotomy due to insertion of the left internal thoracic artery [2], pleural drain insertion [3], diaphragmatic dysfunction due to manipulation of the viscera and reflex dysfunction of the phrenic nerve caused by the use of cold cardioplegic solution [4,5].

Van Belle *et al. *analyzed the pulmonary function of 18 patients before surgery and in the first and sixth weeks after CABG and concluded that respiratory muscle weakness contributed to the decrease in function seen in the first postoperative week. In another study with 37 patients who had undergone CABG, forced vital capacity (FVC) decreased by 70% in the immediate postoperative period and remained reduced in 35% of patients up to 3 weeks after surgery [6].

Many studies have shown the efficacy of physiotherapy, such as incentive spirometry [7] and respiratory muscle training [8], in minimizing pulmonary dysfunction during the preoperative and postoperative periods. However, these studies do not describe the effects on pulmonary dysfunction, especially volume reduction and respiratory muscle strength, over a sufficient period of time. Therefore, the present study aims to evaluate the effect of physiotherapy during cardiac rehabilitation phase I on pulmonary function in patients who underwent CABG.

## Patients and methods

This longitudinal study included 42 subjects aged 48 to 78 years. Subjects were divided into the following three groups: patients who underwent CABG, patients with coronary artery disease (CAD) and healthy volunteers (HV). The CABG and CAD groups were recruited at the Hospital de Cardiologia Procordis.

### Inclusion criteria

We Included for the study were, all candidates for elective coronary artery bypass graft surgery, were recruited and evaluated from January to 1999 to January to 2000, age 40 to 80 years and written informed consent and the study was approved by the institutional ethics committee (Resolution 196/96 of the National Health Council). The three groups were paired by age and gender. The patients included in the CAD and CABG groups were also paired in relation to the number of diseased blood vessels, verified by percutaneous coronary angiography (obstruction≥ 50%). All of the subjects from the CAD group had been previously recommended for CABG but instead chose medical treatment.

### Exclusion criteria

We excluded patients who had a history of previous cardiac surgery, diabetes mellitus, pacemaker implantation, atrial fibrillation, chronic heart failure, utilization of intra-aortic balloon pump, mechanical ventilation longer than 24 hours, acute myocardial infarction within 6 months prior to the surgery, autonomic neuropathy and pulmonary disease.

### Protocol

All the subjects in the CABG group had a preoperative evaluation and orientation in accordance to the physiotherapy procedures. After surgery, they had physiotherapy sessions twice a day for 30 minutes up to postoperative day 6 or until discharge from the hospital (Table 1).

**Table 1:** Physiotherapy protocol after extubation up to postoperative day 6 or hospital discharge

PO day	Physiotherapy protocol
1	CPAP with face mask for 20 minutes, reexpansion respiratory exercises, pursed lips breathing, incentive spirometry, huffing, coughing and transfer patient to an armchair
2	Same as PO day 1 walking around the patient's room
3 and 4	Respiratory reexpansion exercises, pursed lips breathing, incentive spirometry, a 60-meter walk and a walk down 17 stairs with the return upstairs using a lift (PO day 4)
5 and 6	Same as PO day 4, walk 120 meters and walk up a flight of stairs.

PO = postoperative; CPAP = continuous positive airway pressure.

After discharge, all groups received physiotherapy, which included respiratory exercises and walking. They were monitored for 30 days and were evaluated on the following schedules: day 1 for the HV control group, days 1 and 30 for the CAD group, and preoperation and postoperative days 3, 6, 15 and 30 for the CABG group.

### Ventilation test

The pulmonary function tests were performed to measure FVC were measured by spirometry (Spirodoc- Mir; Rome, Italy). For the procedures, subjects sat with their feet resting on the floor. They were asked to inhale deeply to measure the total lung capacity (TLC) and to strongly exhale into the spirometer's mouthpiece to measure the residual volume (RV). Three tests were performed, and the best result was selected. The reference values in the Guidelines for the Pulmonary Function Testing were used for this study [9].

Maximum inspiratory pressure (MIP) and maximum expiratory pressure (MEP) were measured with manuvacuometers (M120 healthcare; 2001; São Paulo, Brazil) to verify static respiratory pressure. The manuvacuometer mouthpiece has 2-mm holes that dissipate the pressures generated by the facial muscles and the oropharynx. To measure the MIP, individuals were instructed to exhale up to the RV, inhale deeply with the manuvacuometer's mouthpiece (Müller's maneuver) in place and maintain the strain with their respiratory muscles for 3 seconds [10].

To measure the MEP, participants were instructed to deeply inhale up to the TLC, do a forced exhalation (Valsalva maneuver) and maintain the strain with their respiratory muscles for 3 seconds.

### Physiotherapy of protocol

On the day preceding surgery, patients received orientation and training regarding the physiotherapeutic procedures to be conducted in the postoperative period, such as re-expansion respiratory exercises, pursed lips breathing, flow and volume incentive spirometry, huffing, holding a cough pillow to the chest and continuous positive airway pressure (CPAP) by face mask. In the first 12 hours after surgery, the subjects were extubated, they received CPAP for 20 minutes and they continued respiratory exercises, such as huffing and directed coughing, from postoperative days 1 to 6 (Table 1).

### Statistical analyses

Statistical analysis was performed in Statistica version 7.0 (Stasoft Corporation, Tulsa, USA). Sample size was calculated and resulted in 10 patients, to achieve an alpha error of 0.05 and power of 0.9. Two-way ANOVA was used for repeated measures. The main factors were time and groups (control, CABG and CAD). When differences were found, the Bonferroni post-hoc analysis was used, and p < 0.05 was considered statistically significant. To compare anthropometric variables in the three groups, single-factor ANOVA (group) was used. Chi-square analysis was used to compare rates of medication use.

## Results

FVC, MIP and MEP were all normal in the HV control group (n = 13). The CAD group contained nine subjects. For the CABG group, 20 patients were recruited and 7 were excluded for the following reasons: diagnosis of lung cancer, pneumonia, study withdrawal and death. The 13 remaining patients (5 females and 8 males) had an average age of 63 ± 2 years (Tables 2). The anesthetic drugs used in the CABG group during surgery were pancuronium, propofol, fentanyl, midazolam and diazepam.

**Table 2:** Anthropometric, clinical and surgical characteristics of each group of subjects

Characteristics	CABG (n = 13)	CAD (n = 9)	Control (n = 9)
Gender (male/female)	8/5	5/4	5/8
Age (years)	64 ± 2	64 ± 2	63 ± 2
BMI (kg/m^2^)	27.6 ± 0.6	27.4 ± 0.7	26.5 ± 0.9
Number of veins	3 ± 1	3 ± 1	
Torrington and Henderson scale (points)	4	4	
Extracorporeal circulation time (min)	85 ± 24		
AC (min)	56 ± 18		
TTOT (hours) Medications	4.2 ± 2.3		
			
ACE inhibitors	5	6	
Digitalis	3	0	
Antiarrhythmic drugs	1	0	
ASA	7	3	
Calcium channel antagonists	4	1	

CABG = coronary artery bypass graft; CAD = coronary artery disease; BMI = body mass index; AC = aortic clamping; ACE = angiotensin-converting enzyme; TTOT = orotracheal intubation time; ASA = acetylsalicylic acid

**Figure 1:**
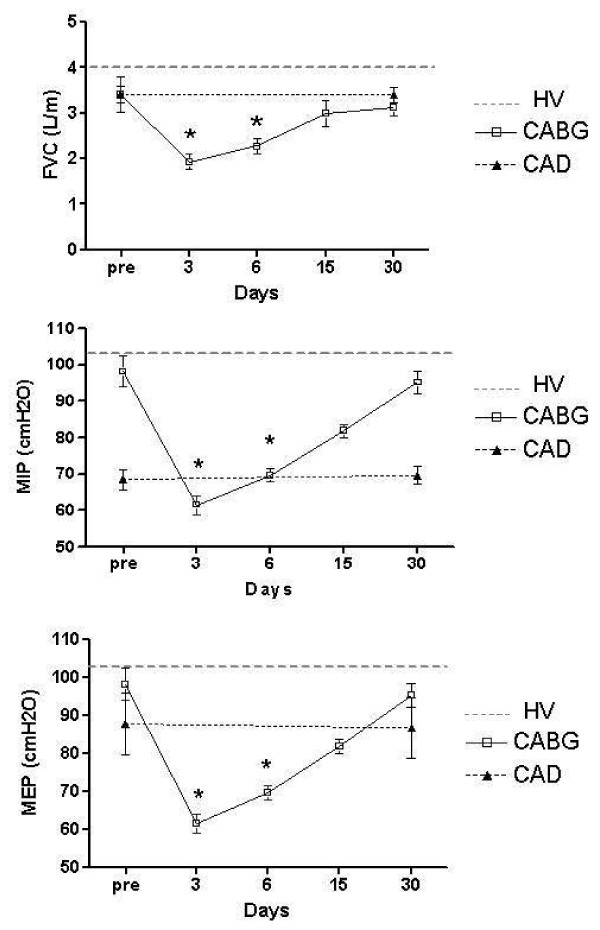
**Forced vital capacity, maximal inspiratory pressure and maximal expiratory pressure in the preoperative period and on postoperative days 3, 6, 15 and 30**. *p < 0.05 pre *vs*. days in CABG. FVC = forced vital capacity; MEP = maximal expiratory pressure; MIP = maximal inspiratory pressure; CABG = coronary artery bypass graft; CAD = coronary artery disease; HV = healthy volunteers

At the first evaluation, there were no significant differences in FVC, MIP or MEP among the groups (p > 0.05) (Figure 1). In the CABG group, there was a statistically significant decrease in the FVC (p < 0.05) on postoperative days 3 and 5. The biggest decrease was on postoperative day 3, and lung function returned to the preoperative level by postoperative day 15 (Figure 1).

The MIP significantly decreased (p < 0.05) in the CABG group on postoperative days 3, 6 and 15 compared to the preoperative MIP. The MIP returned to the preoperative value by postoperative day 30 (Figure 1). The MEP was significantly decreased (p < 0.05) on postoperative days 3 and 6 and returned to the preoperative baseline by postoperative day 15.

## Discussion

Many factors have been suggested to be responsible for the decrease in pulmonary function and consequently FVC [1] and muscular strength after CABG. Some suggested factors include anesthesia, analgesics, surgical stress, pain [11], reduced ventricular function, phrenic nerve injury, cardiovascular drugs, extracorporeal circulation (EC) [12] and the position of the drains [13].

Pain due to thoracotomy is a limiting factor for mobility and breathing. A high level of postoperative pain is common because of the cutting of the skin, muscles and pleura as well as the retraction of muscles and ligaments, pleural and septal nerve irritation from thoracic drains and occasional rib fractures [14].

Yung *et al. *[15] studied the analgesic effect of morphine infusion via spinal catheter in 40 revascularized patients. They found a significant reduction in pain, respiratory insufficiency, extubation time and reintubation percentage compared to the control group. Although analgesia is necessary for shortening mechanical ventilation time and must be given during the first few postoperative hours, analgesia in the postoperative period interferes with pulmonary function. In this study, the analgesic dosing was standardized (Table 2).

Pain may also contribute to decreased cough efficiency, which is the main mechanism for the elimination of secretions from the tracheobronchial tree due to the immobility of the thoracic wall. This immobility causes superficial breathing, which may result in atelectasis, inadequate ventilation-perfusion ratio and pneumonia. These complications lead to increased morbidity [13].

Our results showed a 33% decrease in pulmonary function on postoperative day 3 and a 23% decrease on postoperative day 6 compared to the preoperative period. These results are similar to the existing literature on pulmonary function on postoperative day 6 ([16-20]).

In a study developed by Shernkman et al. with 37 subjects who underwent CABG, there was a 70% decrease in FVC immediately after surgery. FVC remained reduced in 35% of subjects up to 3 weeks after surgery. We can attribute this difference to the physiotherapy that our subjects received up to postoperative day 6 [16].

Left ventricular dysfunction, which increases extravascular fluid in the lungs, leading to altered lung compliance and increased pulmonary resistance, may result in increased respiratory work and oxygen consumption [21,22].

Phrenic nerve injury may also cause a reduction in pulmonary function. Anatomically, the right phrenic nerve travels between the pericardium and the mediastinal pleura, and the left phrenic nerve descends between the subclavian artery and left common carotid lateral to the vagus nerve and anterior to the left lung root. Proposed mechanisms for phrenic nerve injury include decreased nerve conductance due to the freezing of the myocardium in the preoperative period for myocardial preservation [17,22], reduced perfusion of the phrenic nerve due to injury of the branches of the internal mammary artery during dissection [21,23], trauma to the phrenic nerve during sternotomy [24] and internal jugular vein puncture [18,22].

Phrenic nerve injury during heart surgery has an incidence of 26% [23] and may cause diaphragmatic paresis. The right phrenic nerve is more commonly injured, and, rarely, bilateral injury can occur [7].

*Pulmonary function after CABG and physiotherapy *All patients undergoing CABG received physiotherapy from before the operation up to postoperative day 6. Haeffener *et al. *[25] demonstrated the efficacy of positive expiratory pressure (PEP) respiratory incentive spirometry in preventing pulmonary complications after heart surgery. Their sampling was composed of 34 patients who underwent CABG (PEP group, n = 17; control group, n = 17). Their results were similar to the results of this study in relation to the decrease in pulmonary function in the PEP group. On postoperative day 7 there was a significant decrease in FVC, MIP, MEP and 6-minute walking test results, except for subjects in the PEP group. Notably, the PEP group had a lower rate of postoperative complications, such as pneumonia.

Borghi-Silva *et al. *[18] investigated the effects of respiratory physiotherapy with PEP incentive spirometry on phase I cardiovascular rehabilitation, pulmonary function and respiratory muscle strength after CABG. It was concluded that cardiac surgery reduces pulmonary function and the respiratory muscle strength, especially on postoperative day 5. However, the combination of physiotherapy and PEP incentive spirometry reduced these complications. Although PEP incentive spirometry is widely utilized in hospitals before and after CABG, its acute effects on cardiovascular function are not yet clear as the only data in this area are from healthy individuals [26].

Herdy *et al. *[7] investigated the hypothesis that respiratory physiotherapy might minimize adverse cardiopulmonary effects after CABG in 56 subjects (pulmonary rehabilitation group, n = 29; control group, n = 27). Pulmonary rehabilitation was started at least 5 days prior to the surgery, and the protocol adopted, similar to the one used by our group, included CPAP.

Subjects in the pulmonary rehabilitation group had less mechanical ventilation time, atelectasis, pneumonia and atrial fibrillation as well as a shorter hospitalization time.

Various studies have confirmed that this therapeutic modality contributes to decreased PaCO_2_, transpulmonary pressure, respiratory work and hypoxemia and increases pulmonary volumes, mainly FVC, to prevent atelectasis [26,27]. For this reason we used CPAP in our experimental protocol.

Recently, many studies have investigated the benefits of respiratory muscle training [8,27] for postoperative outcomes.

Hulzebos *et al. *[8] developed a randomized, experimental protocol to analyze the inspiratory muscle training (IMT) efficiency for minimizing pulmonary complications after CABG. Their study randomized 279 subjects to IMT (n = 140) or conventional treatment (n = 139). Both groups received the same treatment after surgery.

The protocol was started 10 weeks prior to surgery with IMT being performed 7 times a week for 20 minutes each time. Six sessions were unsupervised, and one was supervised. The protocol was initiated with 30% of the MIP. The IMT group had fewer postoperative pulmonary complications. It was concluded that IMT during the preoperative period minimized pulmonary complications after CABG.

### Clinical implications

In this study, the alterations in pulmonary function and respiratory muscle strength in subjects after undergoing CABG demonstrate the need for early intervention in the preoperative period with the goal of optimizing pulmonary function. Physiotherapy, including incentive spirometry, should be continued in the postoperative period.

### Limitations of the study

The present study should be interpreted in the light of some limitations. First of all, the sample size was small. Second, the intervention was carried out only during the hospitalization period, and the same physiotherapy protocol was utilized for all patients. It must be emphasized that for ethical reasons, it was not possible to have a control group that did not receive treatment. Therefore, this study is not intended to evaluate the effects of physiotherapy on the decrease in pulmonary function after CABG. However, we can infer that the treatment had a positive effect because we observed a lower degree of pulmonary dysfunction compared to that described in the literature.

Third, the time interval among the evaluations was relatively long, especially from postoperative days 6 to 15 and 15 to 30. As a result, we were not able to accurately define when pulmonary function returned to normal values.

## Conclusion

Pulmonary function decreased after CABG. Pulmonary function was the worst on postoperative day 3 and began to improve on postoperative day 15. Pulmonary function returned to the preoperative baseline on postoperative day 30.

## Competing interests

The authors declare that they have no competing interests.

## Authors' contributions

AMM contributed to the study design, literature search, data analysis, manuscript writing and editing. RRTC, MS, PPSS and SLDC participated in the study design, data analysis, manuscript writing and editing. ACLN supervised the study and contributed to the data analysis, manuscript writing and editing. All of the authors read and approved the final manuscript.
